# From ideals to deals—The effect of impartiality experience on stakeholder behavior

**DOI:** 10.1371/journal.pone.0182263

**Published:** 2017-08-07

**Authors:** Marja-Liisa Halko, Topi Miettinen

**Affiliations:** 1 Department of Political and Economic Studies, University of Helsinki, Helsinki, Finland; 2 Department of Economics, Aalto University School of Business, Helsinki, Finland; 3 Helsinki Center of Economic Research (HECER), Helsinki, Finland; 4 Hanken School of Economics, Helsinki, Finland; 5 PCRC laboratory, University of Turku, Turku, Finland; Middlesex University, UNITED KINGDOM

## Abstract

In this paper, we study a two-party pie-sharing problem in the presence of asymmetries in the stakeholders' private endowments. Both the two stakeholders and third-party arbitrators may influence the outcome. We consider Nash-demand negotiations, where the two stakeholders place demands and share the pie accordingly if demands are compatible, and elicit dictatorial allocations from the stakeholders and the arbitrators. The Nash demands by stakeholders are strategic; the dictatorial allocations by stakeholders and arbitrators are non-strategic. We are interested in the influence of the past arbitrator experience on stakeholder allocations and demands and the past stakeholder experience on third-party arbitration allocations. We find that the ex-arbitrators' stakeholder allocations differ more from the impartial ideal than the stakeholder allocations by those without arbitration experience. In contrast with previous findings, the arbitration outcomes do not depend on the asymmetries in the previous stakeholder roles.

## Introduction

Does judgment of fairness depend on the juror’s own position in society? To the least when there are no explicit incentives to act otherwise, are people capable of putting themselves in an impartial position when judging distributional fairness between other people? I.e., is it that on average the judgments of two arbitrators coincide and are independent of the share of the societal cake that each of them receives? Such questions have become increasingly importance from an applied perspective as arbitrator impartiality provides legitimacy for many explicit conflict resolution institutions and also plays an important role when societies rely on the public sector providing a key corrective and supervisory role for market parties. The way previous stakeholder role influences arbitrator judgment and past arbitrator role influences stakeholder behavior is the key for understanding the challenges relating to the revolving door phenomenon. People switch jobs between regulator and the industry, for instance. Many states have implemented cooling-off periods and revolving door prohibitions in order to safeguard impartiality. Are such institutions needed or not?

In this paper, we ask how past experiences in a rich or a poor stakeholder role influence the impartiality of arbitrator decisions. We also ask, and this is our main contribution, how past experiences in an impartial arbitrator role influence stakeholder behavior in the stakeholder role. In our lab-experimental setup there are initial asymmetries in the exogenously assigned endowments of the stakeholders who shall negotiate a division of an additional windfall profit. There is no option for side payments. Thus if parties wish to redress for the asymmetries in initial endowments, they must share the wind-fall profit unevenly. The negotiations are carried out in a standard Nash demand game fashion. The novel feature is that there are also two arbitrators present each of whom proposes how the wind-fall profit should be shared between the stakeholders. Notice however that despite our applied motivation, our aim is not to mimic the institutional settings of the field where strategic dependencies between the stakeholders’ and arbitrators’ decisions confound behavior and impair the capacity to understand whether fairness ideals are biased even there is no strategic incentive for doing so. Therefore we design an experiment where the arbitrator decisions are probabilistically independent and orthogonal from the stakeholder decisions.

There are two competing fairness ideals in this situation: (i) either share the windfall profit equally or (ii) share the sum of the windfall profit and the endowments equally implying that windfall profit itself must be shared asymmetrically. The fairness ideal one endorses might depend on whether the stakeholder has received a high or a low endowment. The one with the smaller endowment might be more willing to compensate for the differences in endowments and thus favor the split-all fairness ideal; the other with a larger endowment might have a stronger tendency for the split-the-windfall ideal [1]. Past experience may influence the fairness perceptions. On one hand, the impartial arbitration decisions or fairness judgments might be biased towards the ideal favorable to one's past negotiator role [2,3]. On the other hand, a past history in the role of an impartial arbitrator might influence the behavior of a stakeholder. Experience from the arbitrator role could advance the capacity for understanding the positions on each side of the table. Bargaining outcomes might thus be more efficient, the stakeholders would fail to reach an agreement less often, or at least the outcomes might differ from the outcomes reached otherwise. We ask how third party arbitrator experience influences stakeholder choices. This is the novel question addressed in this paper. (See also [4] who compare the redress for endowment asymmetries and the willingness to reward kindness and punish unkindness by stakeholders, on the one hand, and impartial spectators, on the other hand.)

We find that arbitrator experience drastically influences stakeholder behavior. The distance between the stakeholder allocations and the impartial arbitrator allocations provides a measure of the gap between stakeholders' (revealed) preferred deals and the unbiased ideals of impartial arbitrators. This gap is larger when stakeholders have arbitration experience. Arbitration experience thus impacts the stakeholder behavior in an asymmetric and self-serving manner. This is surprising and against our initial hypothesis: experience from the impartial arbitration role should render the beliefs about fair entitlements more concordant and, since decision makers wish to avoid cognitive dissonance and maintain consistent self-image, their dictatorial allocations should also lie closer to each other.

In addition to dictatorial allocations (as in [2,5,6,7,8,9], we also let the stakeholders place Nash demands to negotiate a deal (see [10] for a related study with both dictator and ultimatum protocols). We find that rich stakeholders with arbitrator background place lower Nash-demands than rich stakeholders without arbitration experience. The Nash-demands of the poor stakeholders with arbitration experience are not significantly different from those of the poor stakeholders without arbitration experience. Our focus on stakeholder negotiation choices is reminiscent to the design of [11, 12] and [13].

We also conclude with another somewhat surprising finding. The arbitrator decisions of ex-stakeholders, for their part, are not self-servingly biased. To justify the stakeholder behavior, to reduce cognitive dissonance between one's behavior and one's ideals, it is conceivable that arbitration decisions would be biased towards the ideals in the interest of the past stakeholder role as in [2]. We find no evidence of such bias; the surplus allocations of the ex-negotiators are independent of the past stakeholder role, and this evidence is in line with [5,6].

Relatedly, the interest of [14] lies in non-strategic and dictatorial divisions in front and behind a veil of ignorance about the asymmetries in initial endowments. Behind the veil of ignorance people differ in terms of how much they are willing to compensate for the asymmetries: some do not redistribute at all, some others compensate fully to make parties ex-post payoffs equal, and yet some others choose something in between. These differences are also reflected in the decisions made in front of the veil, yet to a minor extent. In addition to the studies above, the focus on third-party allocations is reminiscent to [9] who examine two orthogonal characteristics that impact the degree of impartiality in arbitrator decisions. They find that both a higher payoff- and a higher information-independence induce more redress and thus more ex-post egalitarian outcomes.

There is also an experimental literature on the effect of arbitration on settlement outcomes in conflict situations [15,16,17]. In our experiment, the probability that the sharing proposed by an arbitrator determines the outcomes is independent of whether the Nash demands are compatible or not. To the contrary, in institutional arbitration in real settings, a conventional arbitrator’s [18] proposal only applies if the negotiating parties fail to find a compatible agreement. Our aim is not to mimic real life arbitration institutions but rather merely to study the effect of experience in the impartial arbitrator (stakeholder) role on behavior in the stakeholder (impartial arbitrator) role.

The paper is organized as follows. The experimental design and procedures are explained in detail Section 2. The theory, the hypotheses, and the power tests to determine the sample size are explained in Section 3. In Section 4, we present the results. The final section briefly concludes.

## Experimental design

The experimental sessions were conducted in September 2014 and April-May 2015 at the PCRC laboratory at the University of Turku, Finland. Subjects were recruited using ORSEE software [19] and the experiment was programmed and conducted using the z-Tree software [20]. See S1 Appendix for details of the experimental protocol.

The one-shot game played in each period was as follows. There were four players in the game: one rich stakeholder (player A), one poor stakeholder (player B), and two arbitrators (players C and D). The poor stakeholder had a low endowment of 0 euros and the rich stakeholder had a high endowment of 6 euros. Each arbitrator had an endowment of 6 euros. The task of the two stakeholders (A and B) was to divide an additional 12 euros between themselves. There were five alternative mechanisms that determined how the additional 12 euros were divided between A and B.

The first mechanism was the negotiation game between the stakeholders: the rich and the poor stakeholder. They bargained how to allocate an additional 12 euros between themselves. A Nash demand game [21] was used to model this bargaining game: the two players simultaneously made demands and each player received a pay-off corresponding to her demand (in addition to her endowment) if the demands were jointly feasible. If the demands were jointly infeasible (summing up to more than 12) then all payoffs were zero, and even the endowments of all parties, including those of the arbitrators, were destroyed. We implemented the game this way to make sure that someone who considers a particular distribution as unfair would reach perfect equality in payoffs if he or she decides to reject a proposal. Otherwise a player could decide not to reject since even rejection leads to an unequal distribution of some kind. Admittedly, the downside of the implemented experimental design is that someone might not reject a conceivably unfair deal in order not to inflict a negative externality on innocent third parties. The question of whether and how this design detail influences the results is left for future research.

Conflict payoffs can also be seen as an outside option for the negotiators. This outside option with zero payoffs for all parties can be implemented with almost certainty by making a demand of 12 euros and its probability increases with one’s demand. Generally, from a strategic perspective, good outside options should improve one’s bargaining position, but how much might depend on the equity and fairness principles that negotiators apply [22]. In our case where endowments are destroyed in case of conflict, a high endowment should undermine rather than improve the bargaining position again depending on the fairness rule applied.

The second and the third mechanism was to let the arbitrator C or the arbitrator D, respectively, to decide how to divide the 12 euros between A and B. This is the dictatorial allocation by a non-stakeholder. The task of each arbitrator was to allocate a share of the 12 euros to the poor stakeholder and to assign the residual share of the 12 euros to the rich stakeholder. The assignment was carried out without knowing the actions of the stakeholders or the other arbitrator.

The fourth and the fifth mechanism granted the dictatorial decision of how to split the 12 euros to the stakeholder A or to the stakeholder B, respectively. These are the dictatorial allocations by the stakeholders.

Notice that each of the four *dictatorial* mechanisms grants 6 euros to each of the arbitrators at all outcomes (mechanisms 2–5). Even the first mechanism grants six euros to each arbitrator if the Nash demands are compatible. If not, then each stakeholder and each arbitrator receives 0 euros. In all but one of the cases each stakeholders receives her endowment + the share of the windfall that depends on the actions of the players. The exception to this rule is the negotiation mechanism and its impasse outcome with incompatible Nash demands where all parties receive a payoff of 0.

In the end, an electronic dice-roll by the computer determined which of the five mechanisms became payoff-relevant. The first mechanism was chosen with a 1/3 probability and each of the other four mechanisms with a 1/6 probability. The probability that the *negation* mechanism was used was set twice as high as the probability of the four *dictatorial* mechanisms. We did so since in the negotiation mechanism there were two parties rather than one influencing the outcome. Notice yet that as long as decision maker’s preferences satisfy a standard preference independence axiom (which is implicitly assumed in a vast number of experiments that randomly draw a stage or a task for compensation; see [23] and [24] for insightful discussions about this theme), the actual probabilities should not influence the outcome within each mechanism. Some recent literature yet suggests there could be an effect of some kind if the participants have procedural fairness preferences [25, 26, 27, 28]. Indeed a sophisticated participant might act very generously in one mechanism and very selfishly in another and deem her behavior procedurally fair as a whole. The asymmetries in the implementation probabilities of the mechanisms should reduce incentives for such exploitation of the randomness of the compensation, however.

**Fig 1:**
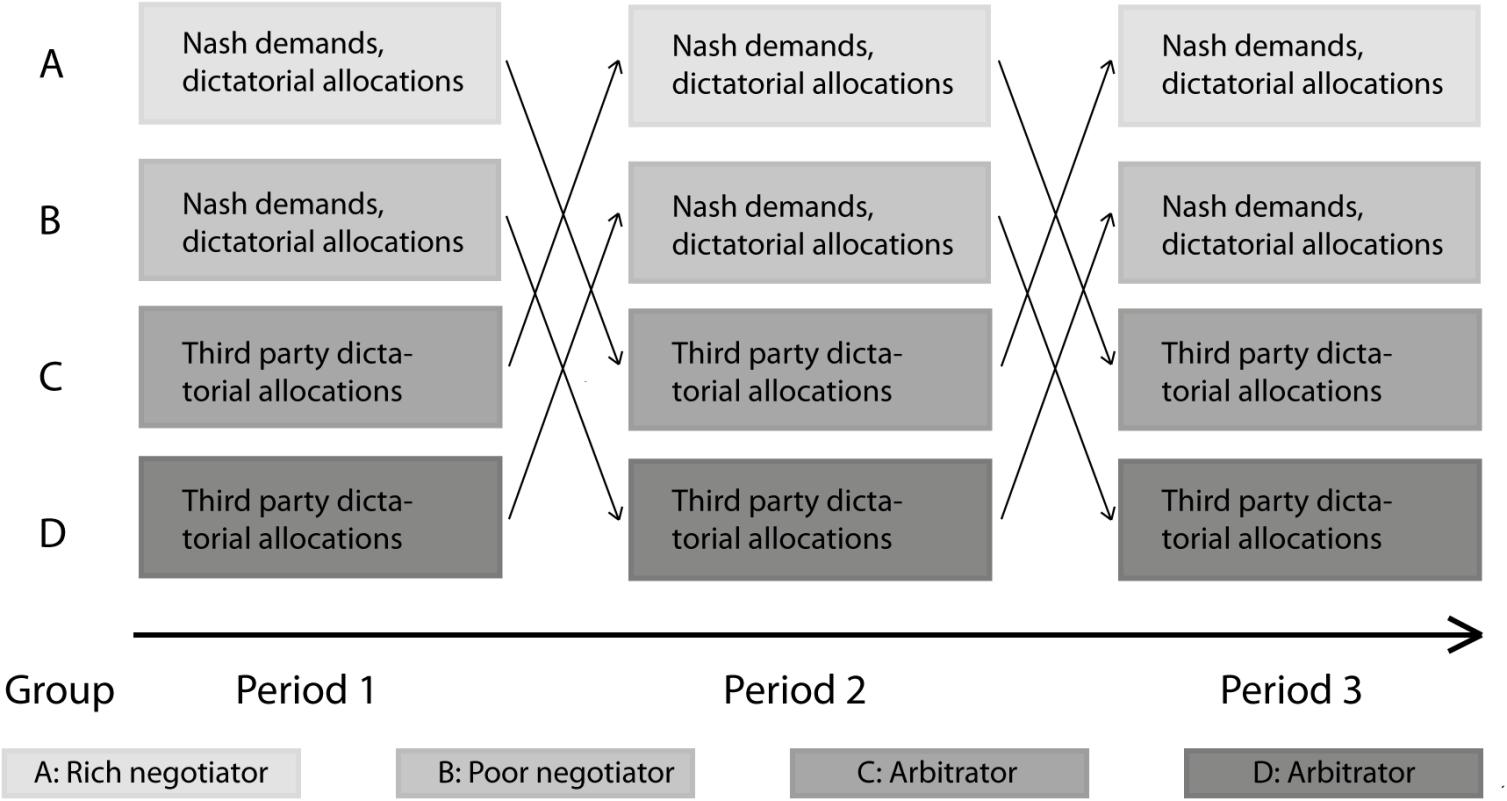
Experimental design. Decisions, timing and role-switching.

The four-player interaction was repeated three times with random re-matching under the restriction that the roles had to be switched from a stakeholder to an arbitrator role and vice versa in each round. Moreover a rich stakeholder in round 1, after turning into an arbitrator in round 2, became a rich stakeholder also in round 3. Similarly, a poor stakeholder in round 1, after turning into an arbitrator in round 2, became a poor stakeholder again in round 3. The timeline and role-switching is illustrated in Fig 1. The role of each participant was announced at the beginning of each round–the participants were not informed of their future roles nor of the repetition horizon (illustrated in Fig 1) at any stage.

Before the experiment started, the participants were randomly allocated one of the three player-roles: a rich stakeholder, a poor stakeholder, or an arbitrator. In each session, half of the subjects started off as arbitrators and half as stakeholders, and again half of the stakeholders started in the rich stakeholder role and the other half in the poor stakeholder role.

Once the game had been played for three rounds, each participant was asked to give her/his best guess regarding (i) the negotiation choice of a randomly chosen rich stakeholder in a randomly chosen round, (ii) the negotiation choice of a randomly chosen poor stakeholder in a randomly chosen round, (iii) the arbitration choice of a randomly chosen arbitrator in a randomly chosen round. The participant was rewarded with one euro for each correct guess. After the elicitation of the beliefs, we used the standard incentivized Holt-Laury procedure [29] to elicit the participants' risk preferences.

The participants learned nothing about the each round’s outcome between the rounds. A random draw by the computer determined which of the three periods was payoff relevant. Each period had an equal 1/3 chance of being chosen. The payoff-relevant round and choice, the outcome of the game, the correctness of the payoff-relevant guess regarding the beliefs, and the remuneration from the Holt-Laury procedure were revealed in the very end of the experiment (this feature excludes learning and repeated game effects). Finally the subjects filled out a post-experimental questionnaire and then each was compensated individually and privately in cash. The average payoff was 14.70 euro including the 3.50 euro show-up fee, and each session lasted between 40 and 45 minutes.

## Hypotheses

The main scope of the experiment is to study whether previous experience in the impartial arbitrator role alters stakeholder behavior. Secondly, we are interested in whether the differential experience in the two alternative stakeholder roles influences arbitration decisions. In this section, we present the key predictions. (See S2 Appendix for the theoretical setup adapted from [2] to our dynamic setting; keeping in mind that the parties are not aware of the future rounds of play and the switching of roles between rounds.) We also present some power calculations based on initial results which ultimately determined our sample size.

### Main hypotheses

Let us first consider our hypothesis concerning the shares allocated to the poor stakeholders by the arbitrators in the second period. We predict that, on average, an arbitrator’s dictatorial allocation is biased towards the respective participant’s dictatorial allocation to the poor in the first period when the participant was involved in the situation in a stakeholder role. This is because in the first period, the belief regarding the fair entitlement may be biased to the direction of the share assigned to oneself to permit grabbing a larger share of the pie without having too much bad conscience about it. At the second round, the belief about the fair share is not equally malleable since the previous round actions and beliefs are freshly recollected. Thus the fairness ideal biased the first round in a stakeholder role influences the perception of how much the poor should be allocated in the second round in the arbitrator role. Thus ex-rich stakeholders who become arbitrators are expected to assign a lower share to the poor than the ex-poor arbitrators. This self-serving bias should drive a wedge between the arbitration decisions in the second period. Yet, in the first period, there should be no difference in the arbitration decisions of the arbitrators in the C and the D roles.

Previous laboratory evidence regarding the existence of self-serving biases in the presence of multiple fairness ideals is mixed. [5, 6] find little or no evidence for self-serving biases in their setting without negotiations, the experimental evidence in [2] is favorable towards the prevalence of self-serving biases and [1] review supportive evidence in negotiation contexts. Finally, the experimental evidence of [11, 13, 30] find supportive evidence in negotiation contexts. Our design studies both negotiation behavior and dictatorial allocations and, in particular, brings into the limelight the question of how past own experiences influence these biases.

In our setting, self-serving biases would imply that (i) the poor are more inclined to consider both the wind-fall pie and the endowments when evaluating fairness, and (ii) the rich are more inclined to consider the wind-fall pie only when evaluating fairness. Thus an arbitrator with experience from the rich stakeholder role allocates less to the poor stakeholder than an arbitrator with experience from the poor stakeholder role. This yields our hypotheses concerning third party dictatorial allocation decisions:

*HYPOTHESIS 1: Arbitrators with stakeholder experience choose dictatorial allocations that are further away from the average impartial fairness ideal (i.e. the average dictatorial allocation of the arbitrators without stakeholder experience)*.

Our design allows us to address a feature not studied previously: how does arbitration experience influence stakeholder decisions? In the first period the arbitrators form opinions about fair entitlements and since they do not hold any stake in the division problem, these should on average coincide with the average impartial views of the fair entitlement. Due to the cognitive dissonance argument, self-deception should be more costly in the second period when the third-party participants become stakeholders. Thus, the self-assigned shares in the dictatorial allocation decisions of the ex-arbitrator stakeholders should be less apart than the dictatorial allocation decisions of the poor and rich who start off as stakeholders. Stakeholders in the rich stakeholder role with arbitration experience should take a lower share of the pie than stakeholders in the rich stakeholder role without arbitration experience and stakeholders in the poor stakeholder role with arbitration experience should take a lower share of the pie than stakeholders in the poor stakeholder and without arbitration experience. This yields out main Hypothesis 2.

*HYPOTHESIS 2: Arbitration experience influences stakeholder decisions (stakeholders’ dictatorial allocations). Stakeholders with arbitration experience choose dictatorial allocations that are closer to the average impartial fairness ideal (i.e. the average dictatorial allocation of the first-round arbitrators) than stakeholders without arbitration experience*.

Up to now, we have considered the effect of arbitration experience on *dictatorial* stakeholder decisions, and vice versa. Obviously, similar effects should apply for the *Nash-demands*. Rich stakeholders with arbitration experience should place a lower Nash-demand than rich stakeholders without arbitration experience and poor stakeholders with arbitration experience should place a lower Nash-demand than poor stakeholders without arbitration experience. This yields out main Hypothesis 3.

*HYPOTHESIS 3: Arbitration experience influences stakeholder decisions (stakeholder negotiation decisions). Stakeholders with arbitration experience choose Nash-demands that are closer to the average impartial fairness ideal (i.e. the average dictatorial allocation of the first-round arbitrators) than stakeholders without arbitration experience*.

### Power tests and priors

The experiments were carried out in two phases. After the first phase in September 2014, when 60 participants had taken part, we investigated the data and studied the observed effects of role switch. Our main hypothesis is that having experience from the third party arbitration role provides a common benchmark for fairness ideals and thus the share assigned to the poor by the rich and poor stakeholders in the second period will be closer to each other and less biased towards the direction of self-interest. Surprisingly, our initial sample of 60 participants showed quite the opposite patterns—the dictatorial allocation decisions of the rich and the poor were more apart when the stakeholders had previous stakeholder experience. The average third party allocation to the poor was 6.97 in the first period. The rich stakeholders without arbitration experience allocated on average 5.44 euros to the poor in the first period but those with arbitration experience allocated on average only 4.79 euros to the poor. Thus the allocations shift away from the impartial ideal by about 0.65 euros—not towards it. The poor stakeholders without arbitration experience, for their part, allocated on average 8.07 euros to themselves in the first period. The poor stakeholders with arbitration experience allocated on average 7.92 euros to themselves. Thus, the arbitration experience does not seem to shift the allocations towards the impartial position on this side of the bargaining table either. In conclusion, if cognitive dissonance plays a role, the stakeholder allocations with arbitration experience should be closer to the impartial allocation than the stakeholder allocations without arbitration experience. We observe the opposite in the initial sample and strive to test for this opposite hypothesis.

We wish to estimate the sample size to have a power of 90% in this one-sided test. Due to the multiple fairness ideals, the allocations to the poor are not normally distributed. Thus we will resort to a Wilcoxon Mann-Whitney U test. We assume that the fractions of allocations observed in the initial sample correspond to those in the true underlying distributions and use Monte Carlo simulations to estimate the sample size. The calculations show that with a sample size of 200 (100 observations without arbitration experience and 100 observations with arbitration experience) yields a power of a bit more than 90%.

## Results

### Preliminary results

Before tackling our main hypotheses, we will first verify some preliminary patterns suggested by existing literature are confirmed in our setting. These constitute a basis for our study. We use first period data to test these basic hypotheses. (The data is available as a supplement in S1 Data.) Later down we test how the behavioral relationships will be influenced by having experience from acting in another role.

**Fig 2:**
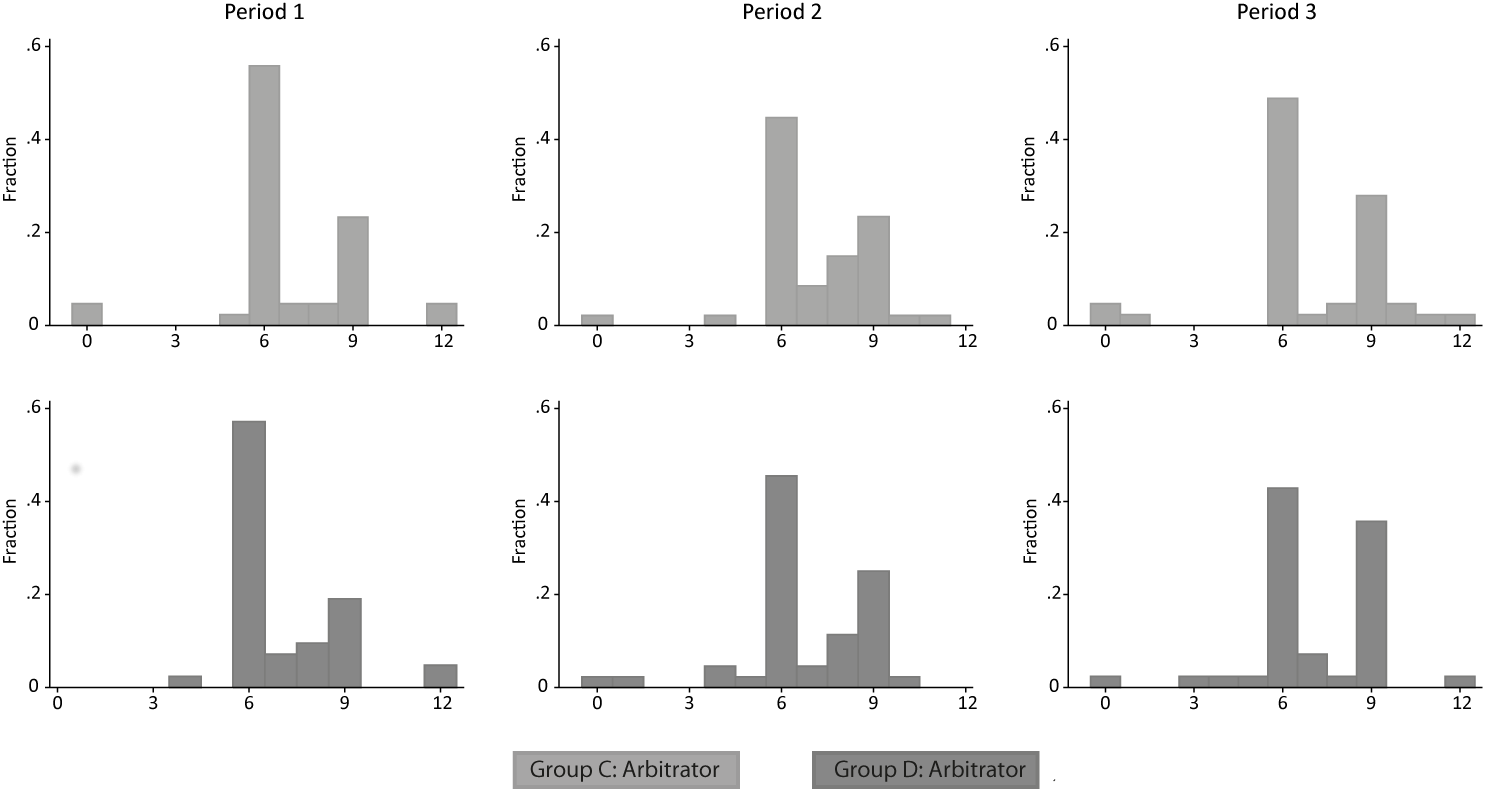
The fairness ideals. The distributions of the shares (out of 12 euro) the arbitrators’ allocated to the poor stakeholder. In every period, the two most popular allocations are 6/6 and 3/9.

The histograms in Fig 2 describe the dictatorial allocations of the arbitrators, in particular the number of euros out of 12 euros that they allocate to the poor stakeholder. Clearly, we can observe two dominant fairness ideals: allocating 6 euros leading to an equal split of the windfall but to unequal total payoffs to the stakeholders, and (ii) allocating 9 euros to the poor stakeholder leading to an unequal split of the pie but equal total payoffs for the stakeholders.

**Fig 3:**
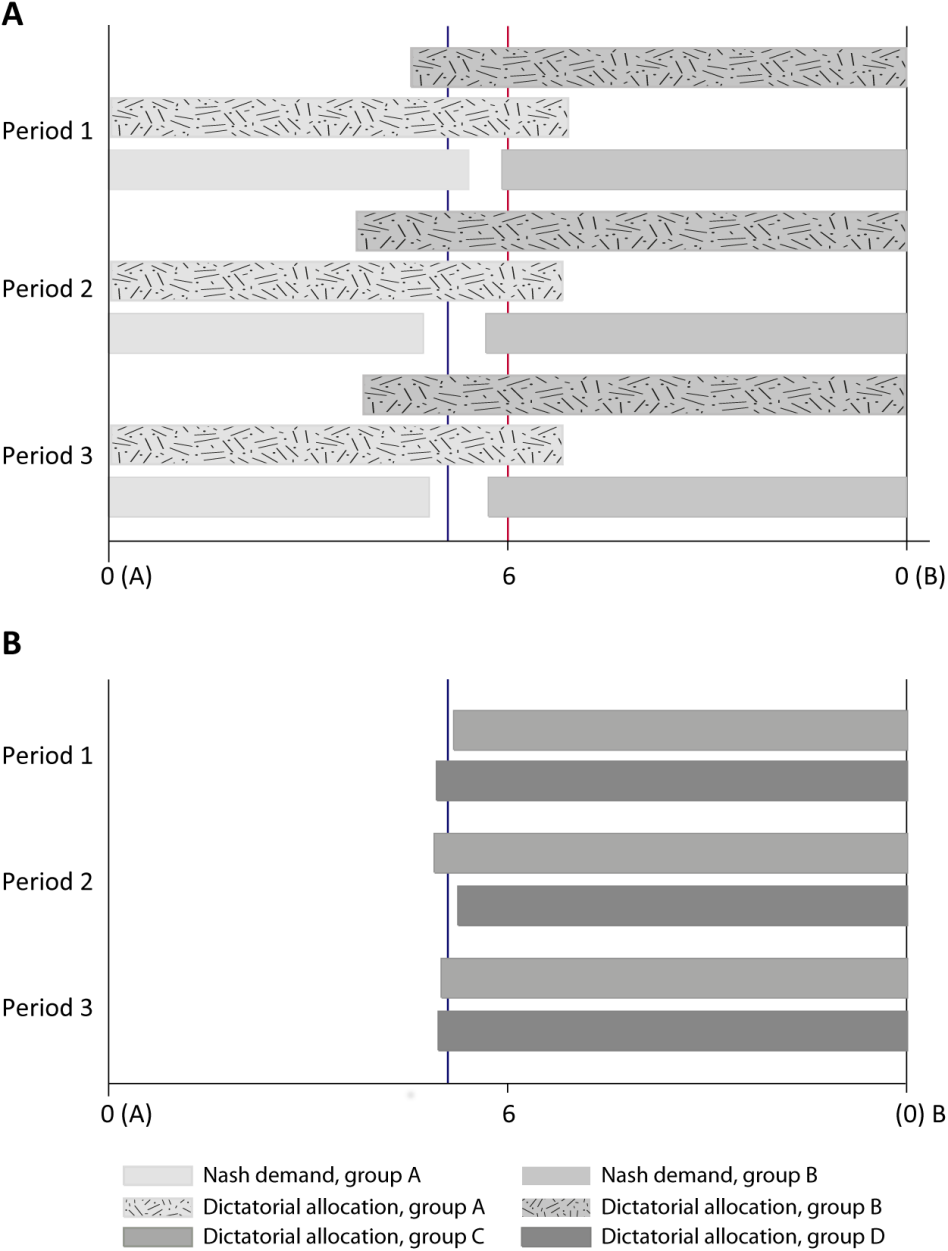
Preliminary results. **Panel A** Stakeholder demands and their dictatorial allocations to self. Rich stakeholders place a smaller Nash-demand than poor stakeholders. (p = 0.067, MW-U; p = 0.013, t-test; one-sided*)* **Panel B** Arbitrators’ dictatorial allocations. In both panels, *η* is the average arbitrator allocation in the first period (6.94 in our sample, N = 98).

Secondly, we can look at the redress for the asymmetries in the endowments. Our findings suggest that there is surprisingly little redress and concern for the worse-off stakeholder in the *negotiation* decisions of the rich stakeholders. Both fairness concerns per se and strategic foresight should impact the incentives of the rich and induce the Nash-demands of the rich to be lower than those of the poor (Table 1). Yet empirically, even if the Nash-demands of the rich are smaller, this difference is significant only at the 10 percent level, or at 5 percent level with specific statistical distributional assumptions (t-test). The periodic averages of the stakeholder conditional on the negotiator role are illustrated in Fig 3, Panel A.

**Table 1:** Descriptive statistics.

Role	Action	Period 1		Period 2		Period 3
Poor stakeholder	Nash-demand	6.07	r	6.31	r	6.27
		(1.21)	o	(1.51)	o	(1.23)
Rich stakeholder	Nash-demand	5.40	l	4.72	L	4.81
		(1.30)	e	(1.53)	e	(1.17)
Poor stakeholder	Allocation to poor / self	7.43		8.26		8.14
		(2.27)		(2.60)		(2.44)
Rich stakeholder	Allocation to poor	5.13	s	5.24	s	5.21
		(2.29)	w	(3.10)	w	(2.67)
	Allocation to self	6.87	i	6.77	i	6.79
		(2.29)	t	(3.10)	t	(2.67)
Arbitrator (ex-poor at 2nd & 3rd)	Allocation to poor	7.07	c	6.75	c	7.05
		(1.70)	h	(2.06)	h	(2.11)
Arbitrator (ex-rich at 2nd & 3rd)	Allocation to poor	6.81		7.11		7.00
		(2.27)		(1.83)		(2.51)

The average Nash-demands and dictatorial allocations by negotiation roles and past experience (standard errors in the parenthesis).

There may be some sign of redress in the strategic negotiation behavior of the rich but once strategic considerations are lifted, there is no difference in how much stakeholders on each side of the table grab to themselves in their respective dictator allocations. Now the rich with high endowments take precisely as much as the poor with low endowments (if there is a difference, it is not significant even at 10% level, Mann Whitney U-test). There could be at least two explanations for these patterns: either fairness does not matter and all stakeholder dictatorial allocations are extremely selfish, or mental accounting is so strong that all of the participants only consider the narrowly fair-minded equal splitting of the windfall. Yet, both of these explanations conflict with the fact that the dictatorial allocations do peak both at 6/6 (splitting the windfall equally) and at 3/9 (splitting the total earnings equally between the stakeholders), corresponding to the two fairness ideals. These findings open up an interesting additional research question related to the main hypotheses 2 and 3: whether the (non)difference in behavior of the rich and the poor will be impacted by arbitration experience. We return to these questions in the following subsection.

While differences in endowments do not seem to have a drastic influence on stakeholder behavior and thus self-servingly biased fairness considerations are somewhat mute, we do find strong evidence for self-interest: *a rich stakeholder allocates a smaller share to the poor than a poor stakeholder allocates to herself (significant at 1% level*, *MW-U)*. We also find support for self-interest being reflected through the understanding of the strategic nature of the negotiations in that strategic bids tend to be lower than dictatorial allocations by the stakeholders: *each stakeholder type's Nash-demand is smaller than her dictator allocation to herself (significant at 1% level)*. There is no strategic risk in the dictatorial decision but strategic risk is present and strategic considerations must be taken in account in the negotiation decisions since too high a Nash demand may result in the pie and the endowments being destroyed. This seems to be well understood by the stakeholders and it suggest that the no-self-serving-bias finding is not due to lack of understanding the strategic underpinnings of the interaction.

### Main results

With some preliminary results at hand, we can proceed and analyze whether and to which extent our main hypotheses are supported by experimental evidence. Notice that given our design with role-switching, our results are between-subjects comparisons.

Let us begin with the third-party arbitration decisions. The periodic averages of the third-party arbitration decisions of agents in roles C and D are illustrated in Fig 3, Panel B. These bars are very consistently of equal width at each period (average across periods 6.53). Thus, we find no support whatsoever for the hypotheses that the third party arbitration decisions would be impacted in a self-serving manner by previous stakeholder experience. This is also confirmed by statistical tests (Hypothesis 1, not significant even at 10% level, MW-U). Such evidence would result if participants behaved somewhat selfishly in the stakeholder role and desired their impartial arbitration decisions to be consistent with their stakeholder behavior. (Notice that the average arbitrator allocation in the *first* period gives the empirical proxy for *η*, the impartial fairness ideal in equation (1) in the Supporting information S2 Appendix. For regression analysis controlling for beliefs about others’ behavior, see S3 Appendix.)

Let's then consider the stakeholder decisions each at a time (see panel A in Fig 3), first the dictatorial allocation decisions and thereafter the negotiation behavior. Regarding the dictatorial choices, we did not find evidence of smaller difference in the dictatorial allocation decisions in each side of the bargaining table. In fact we find evidence of the opposite: dictatorial allocations diverge away from the impartial fairness ideals when stakeholders have previous arbitration experience. The difference between the first-column entries in row 3 and row 4 of Table 1 is significantly different from the difference in the second-column entries in row 3 and row 4 (between-subjects MW-U test). We do not find any evidence of a difference triggered by role-switching from arbitrator to stakeholder on either side of the bargaining table, studying each side separately. The differences between the entries in row 4 of Table 1 and the differences between the entries in row 5 are significantly different from each other (between-subjects MW-U tests). The no-result of impact of arbitration on the dictator choices holds both for the rich and the poor.

*THE OPPOSITE OF HYPOTHESIS 2 HOLDS TRUE: Stakeholders with arbitration experience choose dictatorial allocations that are further away from the average impartial fairness ideal (i.e. the dictatorial allocations of the first-round arbitrators) than stakeholders without arbitration experience (p-value equals 0.026 with a two-sided MW-U test)*.

When it comes to the Nash demands, we do not observe that Nash demands of the ex-arbitrators would be *closer to the average impartial fairness ideal* (Hypothesis 3, not significant even at 10% level, a between-subject MW-U test whether the difference between the first-column entries in row 1 and row 2 of Table 1 is significantly different from the difference in the second-column entries in row 1 and row 2). Yet, arbitration experience does impact the negotiation choices of the rich: Rich stakeholders demand a lower share of the pie than rich stakeholders without arbitration experience (p = 0.013, MW-U; 0.024 t-test). Consequently, the negotiation choices of the rich are closer to the impartial entitlement among those who have arbitration experience. The Nash-demands of the poor are not impacted by arbitration experience (not significant even at 10% level, MW-U).

Although the Nash-demands of the poor are higher than those of the rich, they are not significantly so: we found only weak support of a difference in the Nash-demands of the rich and poor. (For regression analysis controlling for beliefs about others’ behavior, see S3 Appendix.) Moreover we found no significant *difference in the self-assigned share of the rich and the poor* when it comes to the dictatorial stakeholder allocations. As noted in the end of section 4.1, this raises the question of whether the difference in the self-assigned shares of the rich and the poor, on the one hand, and the Nash-demands of the rich and the poor, on the other hand, might turn significant once stakeholders have arbitration experience. Indeed, after role-switching these differences turn highly significant.

*ADDITIONAL RESULT 4a: Arbitration experience influences the difference in stakeholder dictatorial allocations across the roles. A rich stakeholder without arbitration experience takes as much as a poor stakeholder without arbitration experience (p-value of two-side test of difference yields p = 0.16, MW-U). Yet, the allocations of the poor and the rich stakeholders differ when considering stakeholders with arbitration experience (p = 0.019 in period 2 and p = 0.006 in period 3, MW-U)*.

*ADDITIONAL RESULT 4b: Arbitration experience influences the difference in stakeholder Nash demands across the roles. A rich stakeholder without arbitration experience places as high a Nash-demand as much as a poor stakeholder without arbitration experience (p-value of two-side test of difference yields p = 0.067, MW-U). Yet, the demands of the poor and the rich stakeholders differ when considering stakeholders with arbitration experience (p<0.0001, MW-U)*.

We can complement Result 4 by conducting a difference-in-differences test. We run a linear regression where arbitration experience is interacted with role when explaining allocation decision and the Nash-demands, respectively (Supporting information, S1 Table). This reveals that the change in the difference in the Nash- demands (4b) of the rich and the poor is significant at 5% level such that the gap in the demands of the poor and the rich gets wider when the stakeholders have arbitration experience. The gap in the self-assigned share also increases (4a), but this change is not statistically significant.

## Discussion

In this paper, we study bilateral pie-sharing in the presence of asymmetries and a plurality of fairness ideals. There are stakeholders and arbitrators. The stakeholders take two types of choices; strategic Nash-demands in the negotiations and dictatorial non-strategic allocations. The arbitrators share the pie between the two stakeholders in a dictatorial and non-strategic manner. There are thus essentially three mechanisms by which the pie may be divided between the stakeholders: (i) stakeholder negotiations, (ii) stakeholder dictators, (iii) arbitrator dictators. The game is played for three rounds and we are interested in the influence of third-party experience on stakeholder choices and the differential influence of stakeholder experience on arbitration choices.

We expected to observe self-serving biases in stakeholder choices and that these biases would be of smaller extent among stakeholders with arbitration experience. Likewise, we expected that previous stakeholder experience would bias arbitrator choices to the direction of one's stakeholder choices. Our evidence fails to support these hypotheses. In fact regarding the former, our evidence supports the opposite prediction that experience from an impartial arbitration role makes the dictatorial allocations more biased to the direction that benefits the allocating stakeholder. Remark also that somewhat relatedly, [31] finds order-effects in redress behavior when comparing no-veto-cost and ultimatum game protocols.

Although our setup has more similarities than differences with [2], the experimental designs differ in a number of aspects. Most importantly, we do not have treatments where the pie to be shared would be generated in a real-effort task. Such a design might generate a stronger tendency for a conflict in fairness ideals regarding how the pie should be shared. Indeed, [2], alongside [32] among others found less evidence of self-serving biases in treatments where there was only exogenously generated variation in the entitlements (as in our paper). In that case participants were more concordant that uncontrolled inequality, which parties cannot be held responsible for, should be redressed in dictatorial allocations and the weaker party should be compensated. For experiments showcasing the importance of earned entitlements, see [10, 31, 33, 34, 35, 5, 36, 37, 38]. The effects of asymmetries in endowments appear complex. [32] observe that patterns depend not only on whether asymmetries are generated exogenously or endogenously in the lab but also on asymmetries outside the laboratory.

In our experiment, we have only treatments where there is such exogenous inequality and thus indeed third-party allocations should be expected to compensate for it independently of previous stakeholder experience. Thus a potential explanation for our results is that stakeholders without arbitration experience neglect any need for redress for exogenous and random differences in endowments but arbitration experience raises the awareness of such needs and the poor ex-arbitrators in the stakeholder role, in particular, then react to this raised awareness by perhaps self-servingly redressing more than the poor stakeholders without arbitration experience. This is also consistent with the fact that even the stakeholders with both arbitration experience in addition previous stakeholder experience also act this way. In every case, it is clear that in follow-up studies one should examine whether the observed unexpected effects also hold for earned entitlements and asymmetries in these.

Another key difference between our design and virtually all of the rest of the literature designs is that we have a decision screen where all choice problems of all the influential parties are presented simultaneously and the dice roll that determines the payoff-relevant one among all the actions is very vividly illustrated. Thus the design has features akin to the hybrid design of [39] (see the decision screen in the S1 Appendix). The dice roll implements the widely applied randomized incentive protocol to pick up one payoff-relevant action among the many that each decision maker takes. This is done in order to rule out income effects, hedging [24] and the like. In all such experimental designs including the ones where one round is drawn for random payment, there is an implicit Archimedian assumption (independence of irrelevant alternatives) made which ensures that the preferred choice of the agent is independent of the probability of the payoff-relevance of the particular choice task and the outcomes in other tasks. Due to the structure of our decision screen where the dice roll is very explicit, the probabilistic payoff-relevance is highly salient and may thus account for the fact that our choice patterns look rather different. To provide an example: a rich stakeholder may over-shade her bid downwards thus benefitting the poor stakeholder, and this may justify the choice of not compensating the poor in the dictatorial allocation—over-shading suffices to increase the expected balance between the two stakeholder's payoffs (a suitably crafted other-regarding preference model that combines of efficiency concerns [40, 41] and procedural fairness [26, 27] would predict such patterns). Thus, although only one of the stakeholder’s two actions may be payoff relevant at a time, the rich stakeholder may not treat her two actions as independent. (See also [28].)

Notice that our effect of arbitration experience on stakeholder allocations is reminiscent to the findings of [6] who find that an appeal for moral reflection impacts the non-strategic and dictatorial redress of differences in productive activities by stakeholders when the joint returns to those activities are shared. As in [6], the sample consists of Nordic university students. In our case there is no explicit appeal but it is imaginable that the stakeholders with arbitrator experience did engage in moral reflection when acting as third-party arbitrators. The puzzling feature is that this effect seems more pronounced among the ex-arbitrators who end up in the poor stakeholder role. It is obviously more in their self-interest to redress more for the asymmetries. We do not observe similar change in the behavior of the rich stakeholders.

How should we think about our contribution affecting our posteriors regarding the effect of arbitration experience on stakeholder behavior? The question is new but related to earlier research on the effect of stakeholder experience on fairness ideals. How should those earlier contributions influence our priors? We ourselves started with strong priors well above 50%, but given the earlier mixed results concerning the prevalence and existence of self-serving biases driven by cognitive dissonance and contextual inertia of fairness ideals [42]? Given the earlier mixed evidence of self-serving biases in the literature [1, 2, 5, 6, 13], we consider a range of prior probabilities (from 30% to 90%) that the stakeholder allocations of the rich and the poor would be closer together when they have arbitration experience from the impartial arbitrator position. The post-study probability of each of our hypotheses [42] falls to 4,3% with a prior of 30% and to 48,6% with a prior of 90% given the power of 90% and the significance of 5%. Evidence confirming our hypothesis would have updated a prior of 30% up to 88,5% and a prior of 90% to 99,3%.

Though related studies exists and existing theories suggest theoretical predictions, we are not aware of previous experimental research studying the effects of past arbitrator experiences on negotiation and outcomes. The question is novel and interesting. In wage negotiations between unions and in settlement negotiations in legal disputes, for instance, it is professional negotiators who act on behalf their clients and who have variant degrees of experience from the various roles around the negotiation table in similar situations. It is of interest to understand which kind of a negotiator experience is likely to deliver a favorable negotiation outcome both from the perspective of an individual client and from the societal perspective of efficiency or allocative fairness. In this paper we have taken the first modest steps towards a better understanding of some of these factors. To gain a better confidence and wider understanding of such effects, further and complementary field and laboratory behavioral data and surveys are needed.

## Supporting information

S1 Appendix Experimental design *and* instructions. (DOCX)

S2 Appendix Theory. The theoretical setup is adapted from Konow (2000) to our dynamic setting.
(DOCX)

S3 Appendix Supportive evidence and regressions. (DOCX)

S1 Table Difference-in-differences tests. Linear regression models on stakeholder’ Nash-demands and dictatorial allocations in the first two periods.
(DOCX)

S1 Data (XLSX)
