# Supplementary material for: From ideals to deals—The effect of impartiality experience on stakeholder behavior
Source: PLoS One. 2017 Aug 7;12(8):e0182263. doi: 10.1371/journal.pone.0182263 (PMC5546632; doi:10.1371/journal.pone.0182263)
Supplement: S1 Appendix — (DOCX) [file pone.0182263.s001.docx]

**Halko, Marja-Liisa & Miettinen, Topi: From ideals to deals - the effect of impartiality experience on stakeholder behavior**

**S1 File. Experimental design and instructions.**

**Experimental design**

The experimental sessions were conducted in September 2014 and April-May 2015 at the PCRC laboratory at the University of Turku, Finland. Subjects were recruited using ORSEE software [Greiner, 2015] and the experiment was programmed and conducted using the z-Tree software [Fischbacher, 2007]. There were altogether 9 sessions which took place in a 20 seat dedicated laboratory. There were 16 participants in one of the sessions and 20 participants in the remaining eight sessions.

The experiment was not accepted an institutional review board, since Hanken School of Economics did not have one at the time when the experiment was run. Yet, the experiment complies with the international standards of experimental economics research. The participants did not learn the identity of the other participants they interacted with and the identity of the participants cannot be inferred from the data which is entirely anonymous. The participants sign a written informed consent when they join the experimental participant database at the PCRClab.

We checked the identity of the subjects and randomly allocated each a visually isolated cubicle in the laboratory. The participants received a hard-copy of the instructions, written in Finnish (translation in the chapter Instructions). The instructions were read out loud by the experimenter once the participants had read the instructions privately and quietly. Thereafter the experiment was started. First the participants answered three control questions (translation in the chapter Control questions) regarding the four player interaction. They could proceed to the actual experiment only once all three questions were answered correctly. Once all decisions were finished subjects were paid individually according to their choices. The currency for final payments was euros.

**Instructions (translated from Finnish)**

**General instructions**

Many thanks for taking part to this experiment. You will receive 3.50 euros for showing up on time. You can earn even more but this will depend on your choices, those of other participants, and to some extent, on random events. Your responses will be strictly confidential, meaning that your name will never be associated with your test results.

Please, read through the instructions carefully. All participants have received identic

al instructions. Shut down your mobile phone. Notice that you are not allowed to talk to other participants during the experiment. If you have any questions, please, raise your hand. We will answer your question personally.

**Description of the experiment**

You are in a decision making situation with three other participants, but you will not learn the identity of the other three (the others will not learn your identity either). Two of the four participants, A and B, are assigned the role of a negotiator, and the two others, C and D, are assigned the role of an arbitrator. The experiment consists of three rounds. At the beginning of each round, each participant will be in one of the four roles. In each round the negotiators and the arbitrators are altered, and both the negotiators and the arbitrators are randomly re-matched. As a negotiator, you will never be matched with the same negotiator twice.

**During each round, you will make several decisions: the number of decisions will depend on your role during the round in question.**

(1) The task of the Negotiators A and B is share 12 euros between themselves. Simultaneously each of the Negotiators, A and B, states a demand without observing the demand of the Negotiator in the opposing role or the actions of the Arbitrators C and D. A demand is an integer amount between 0 and 12 euro. The payments of the Negotiators and Arbitrators depend on the Negotiators' decision as follows:

**Case 1:** The demands of A and B are not compatible in the sense that they sum up to more than 12 euros, the amount to be shared. In this case, the sharing is automatically rejected. In the case of such automatic rejection, all parties (A, B, C and D) receive 0 euro.

**Case 2:** The demands of A and B are compatible in the sense that they sum up to 12 or less. In this case, each participant receives the share she/he demanded. If the demands sum up to less than 12 euros, the residual will not be paid to either participant. If the negotiators reach an agreement, each party gets *a contract bonus*. The sizes of the contract bonuses will appear on the computer screen during the experiment.

(2) In addition to the demands of the Negotiators A and B, both the Negotiators A and B and the Arbitrators C and D will make so called *sharing decision*. In the sharing decision, all parties propose a division of 12 euro between A and B. The Negotiators and the Arbitrators do not know each other’s sharing decisions, or the demands of A and B.

**In the end of each round, the payments of the round are determined by throwing dice:**

- If the result is 1 (with probability 1/6), *the sharing decision* of the Negotiator A is used to divide 12 euros, in addition, all parties get their contract bonuses.
- If the result is 2 (with probability 1/6), *the sharing decision* of the Negotiator B is used to divide 12 euros, in addition, all parties get their contract bonuses.
- If the result is 3 or 4 (with probability 1/3), *the negotiation decision* of the Negotiators A and B is used as follows:

**Case 1:** The demands of A and B are not compatible in the sense that they sum up to more than 12 euros, the amount to be shared. In this case, the sharing is automatically rejected. In the case of such automatic rejection, all parties (A, B, C and D) receive 0 Euros.

**Case 2:** The demands of A and B are compatible in the sense that they sum up to 12 or less. In this case, each participant receives the share she/he demanded. If the demands sum up to less than 12 euros, the residual will not be paid to either participant. If the Negotiators reach an agreement, each party gets *a contract bonus*. The sizes of the contract bonuses will appear on the computer screen during the experiment.

- If the result is 5 (with probability 1/6), *the sharing decision* of the Arbitrator C is used to divide 12 euros, in addition, all parties get their contract bonuses.
- If the result is 6 (with probability 1/6), *the sharing decision* of the Arbitrator D is used to divide 12 euros, in addition, all parties get their contract bonuses.

**Your payoff**

In the end of the experiment (all rounds), the computer chooses randomly *one* round for actual payment, each round with equal likelihood. The outcome of that randomly chosen round determines your payment. In addition, you will receive 3.50 euros for showing up.

*Please be patient. We are waiting until all participants have read the instructions.*

**Control questions (translated from Finnish)**

Q1: Let us assume that

- B demands 7 euros
- A demands 5 euros
- C assigns 4 euros to negotiator A and 8 euros to negotiator B
- D assigns 8 euros to negotiator A and 4 euros to negotiator B

The contract bonus for each party is 3.

What is the monetary remuneration for player A, if the decision of C is randomly chosen to determine the remuneration?

Q2: Let us assume that

- B demands 7 euros
- A demands 5 euros
- C assigns 4 euros to negotiator A and 8 euros to negotiator B
- D assigns 8 euros to negotiator A and 4 euros to negotiator B

The contract bonus for each party is 3.

What is the monetary remuneration for player A, if the negotiation outcome is randomly chosen to determine the remuneration?

Q3: Let us assume that

- B demands 7 euros
- A demands 6 euros
- C assigns 4 euros to negotiator A and 8 euros to negotiator B
- D assigns 8 euros to negotiator A and 4 euros to negotiator B

The contract bonus for each party is 3.

What is the monetary remuneration for player A, if the negotiation outcome is randomly chosen to determine the remuneration?

**Screenshots (with translations for the main decision screen)**

*
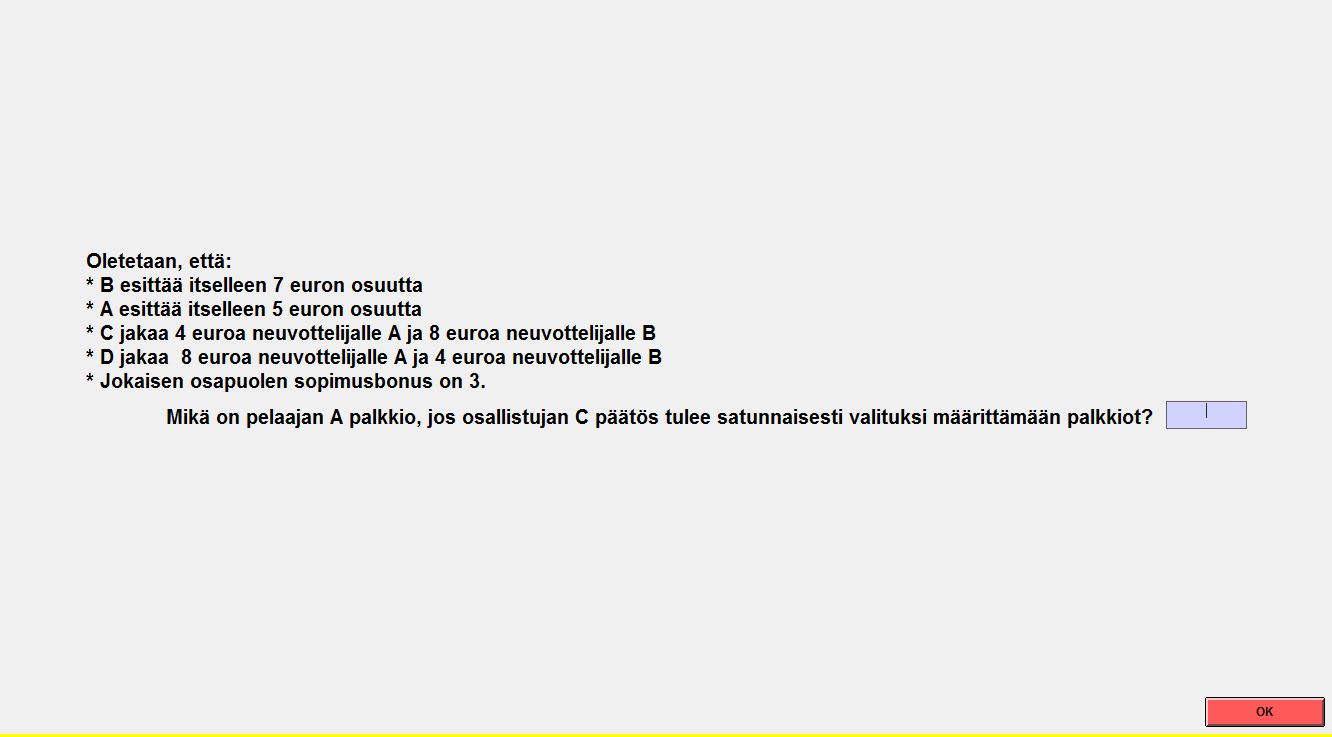
*

**Figure S1:** Control question screen.

*
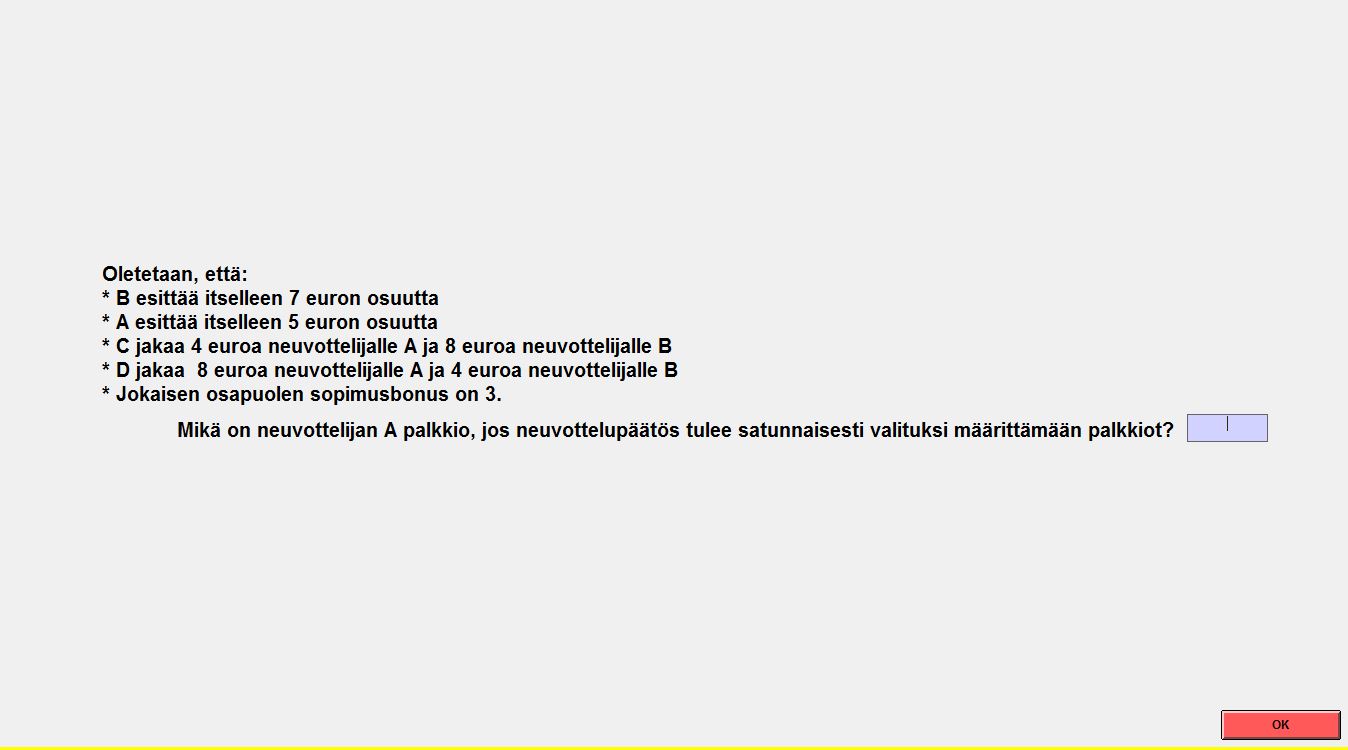
*

**Figure S2:** Control question screen 2.

*
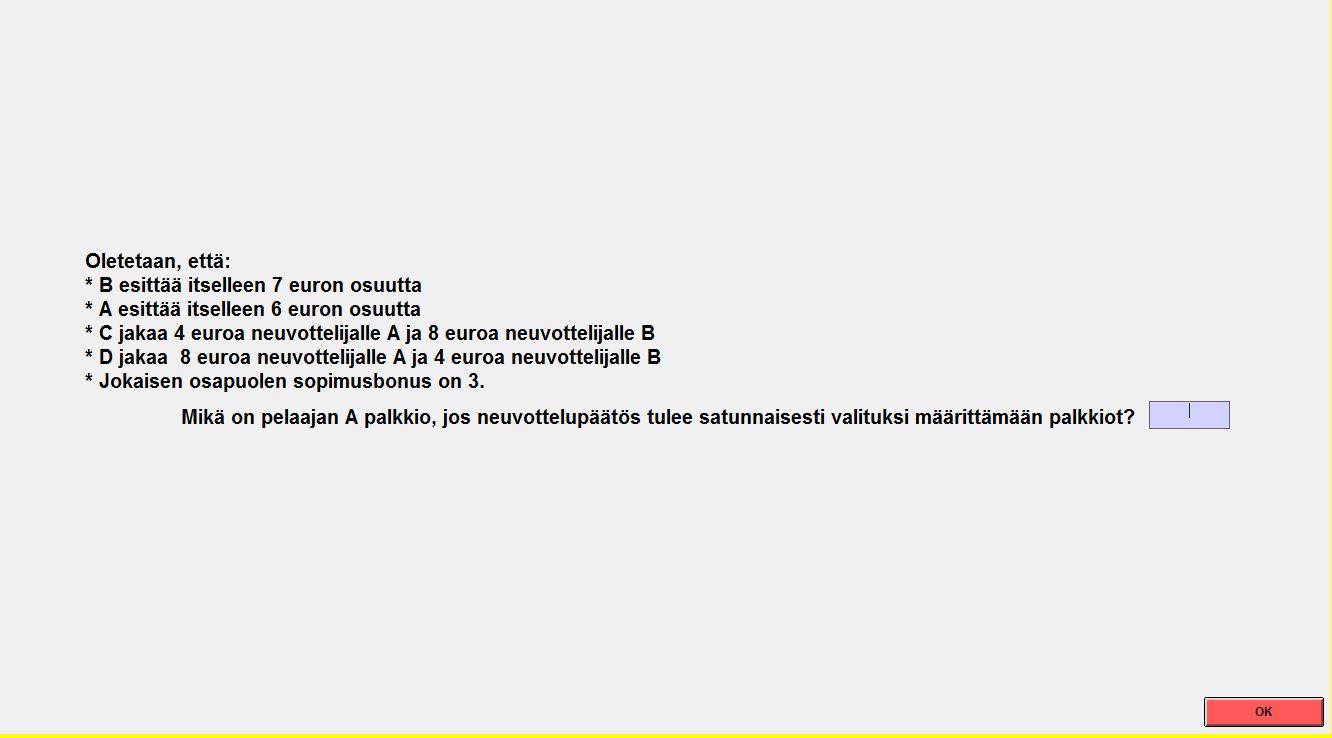
*

**Figure S3:** Control question screen 3

*
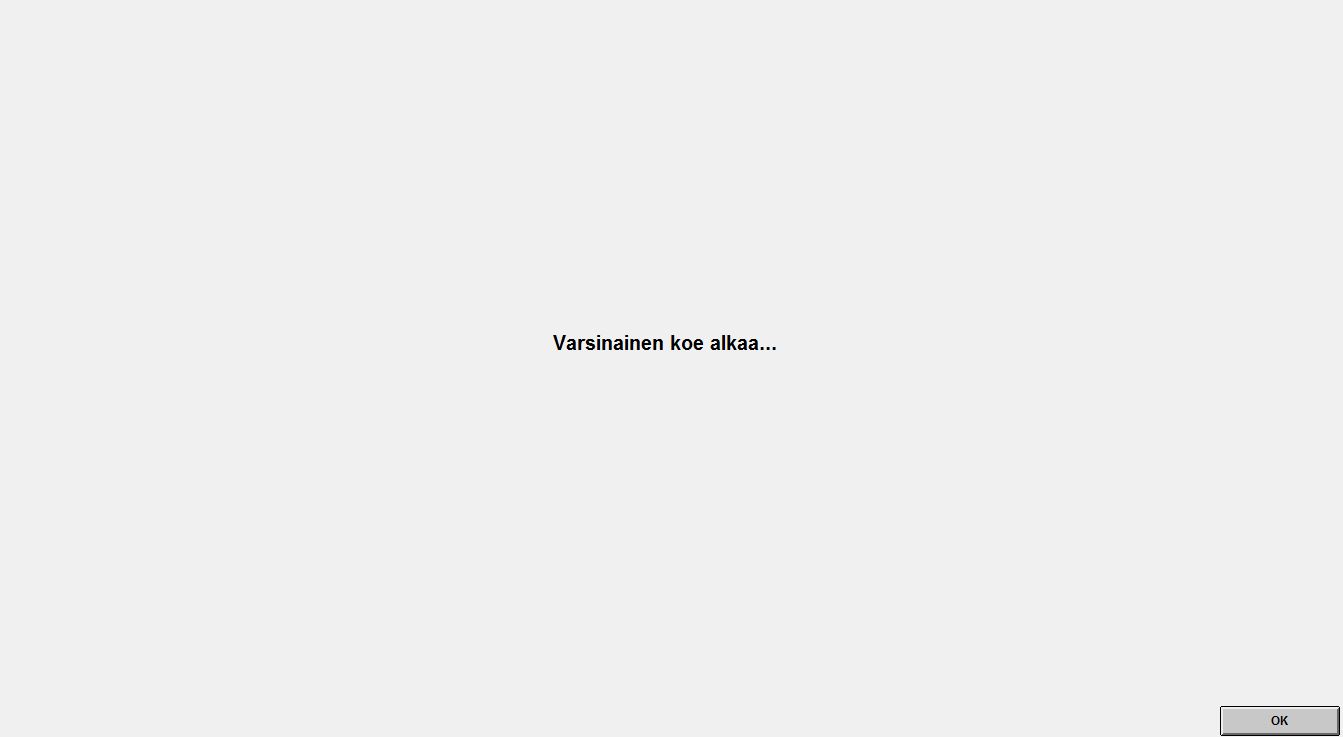
*

**Figure S4:** Waiting screen before the start of the actual experiment.

**
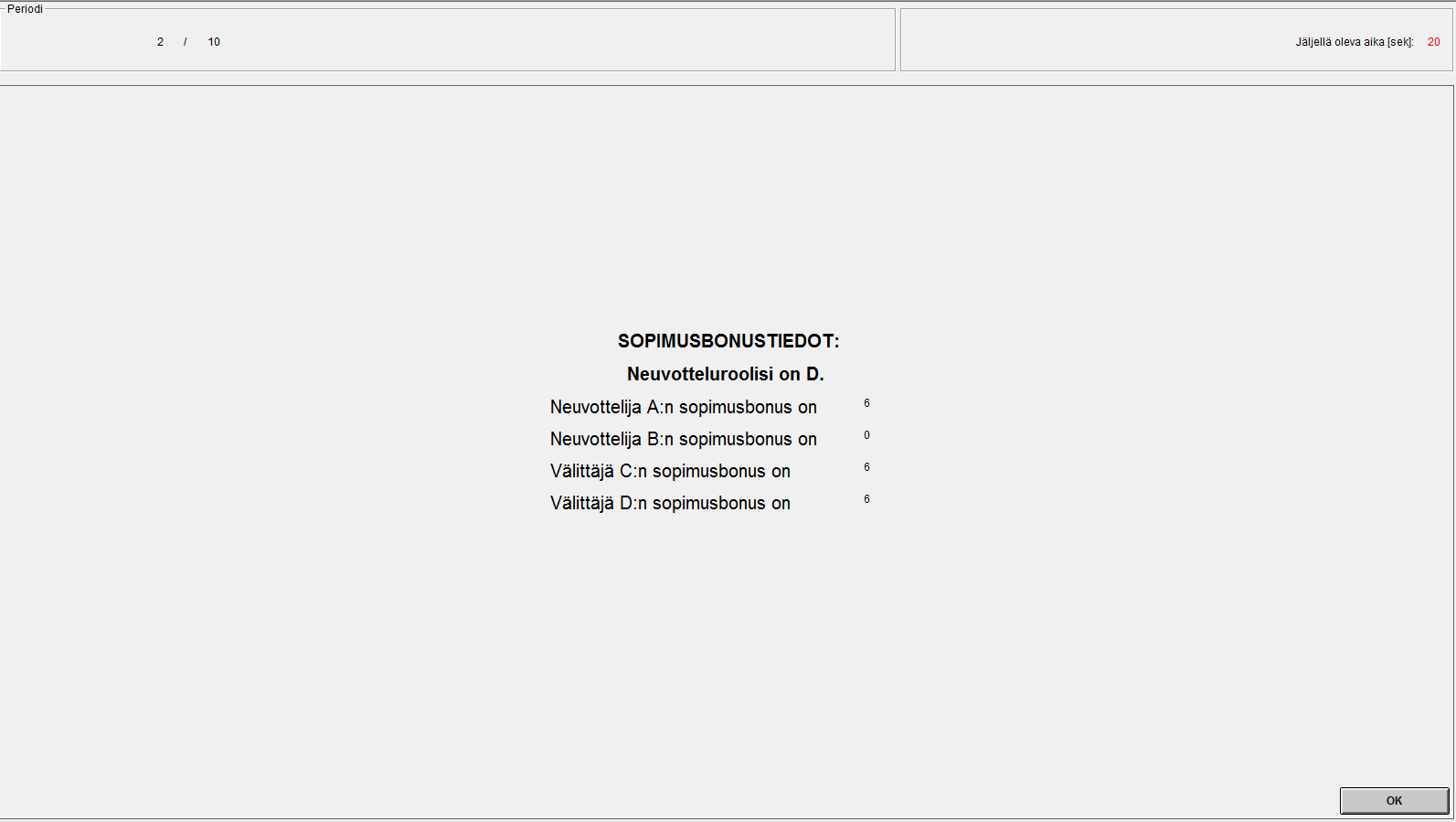
**

**Figure S5:** Role announcement & contract bonus announcements for each role.

**
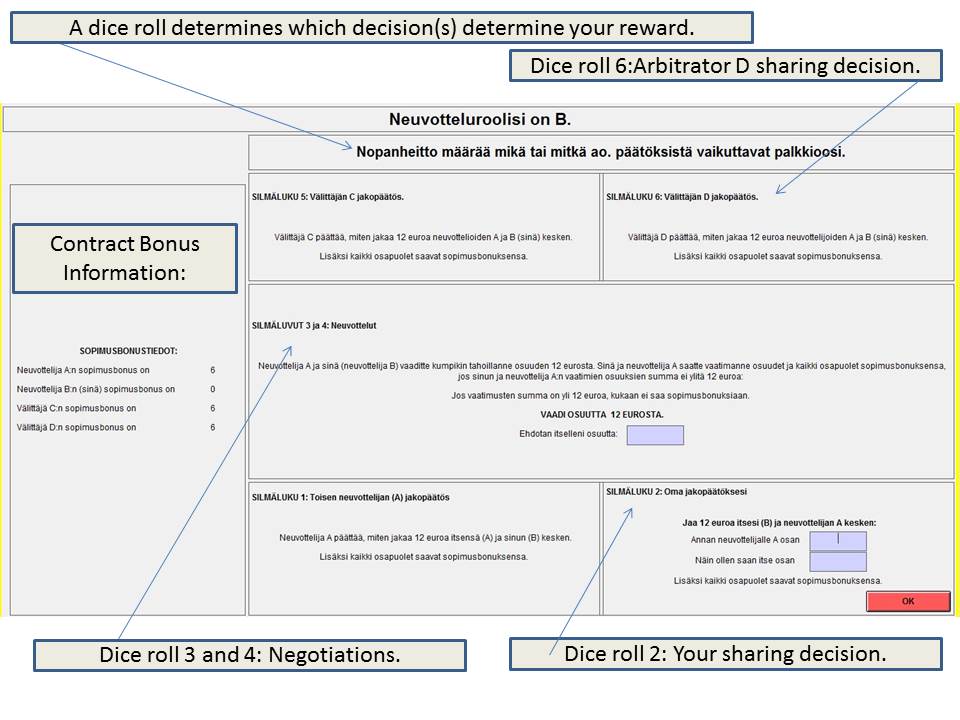
Figure S6:** Main decision screen for a player in role B.

**
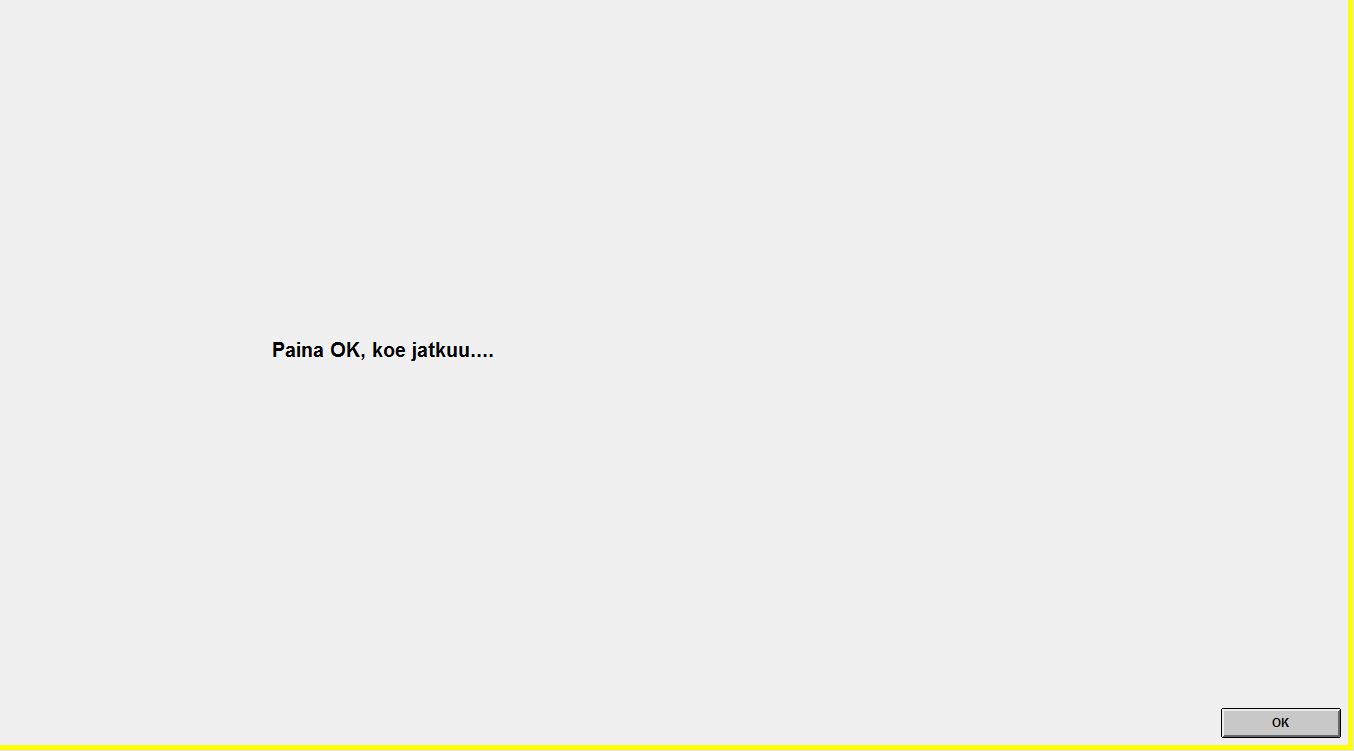
**

**Figure S7:** Interim screen.

**
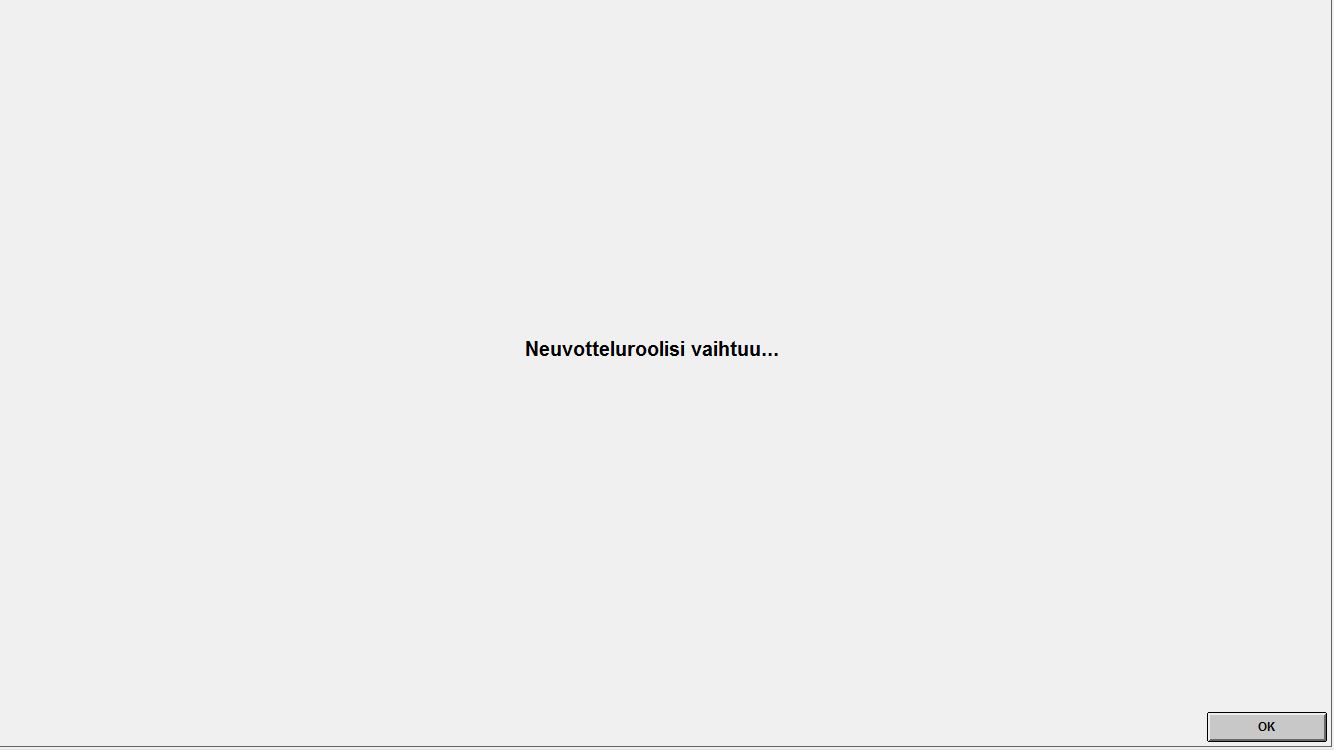
**

**Figure S8:** Role switch announcement screen

**… (Screens S5 to S8 with new roles) …**

**
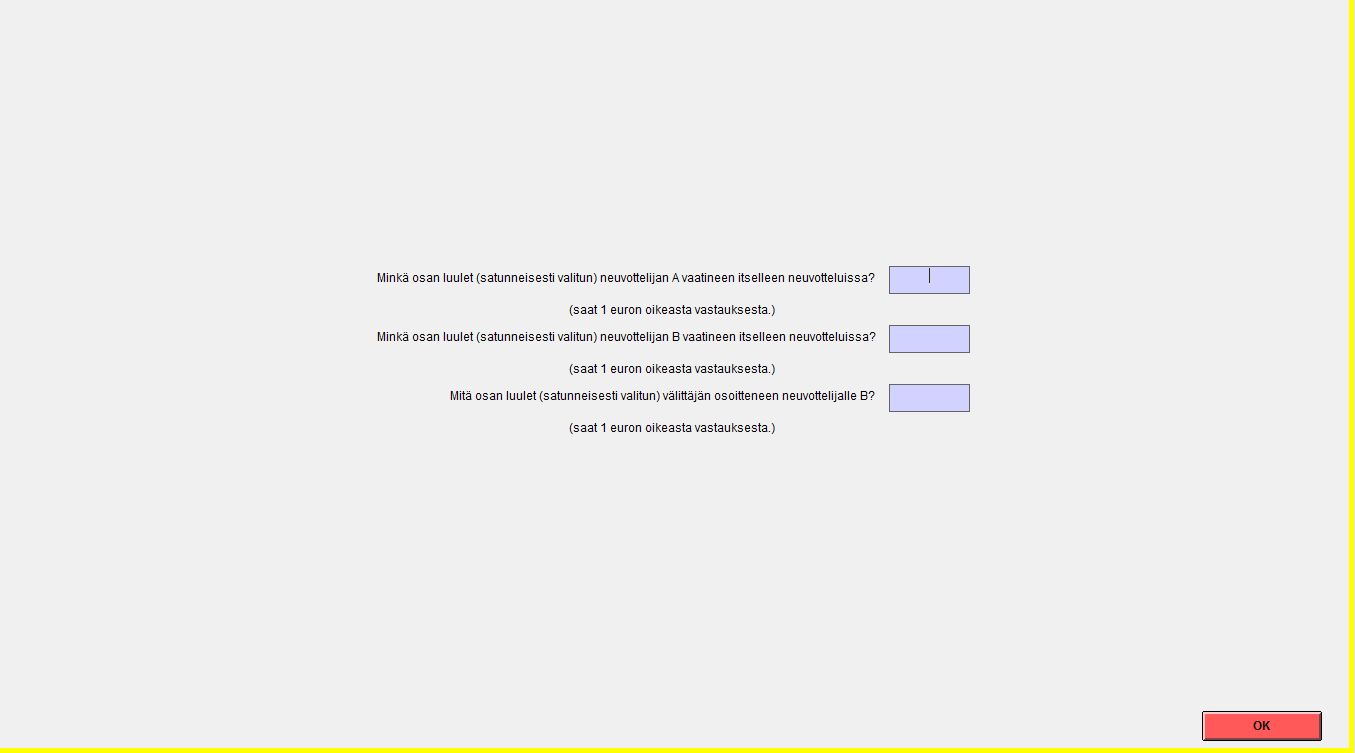
**

**Figure S9:** Belief elicitation screen


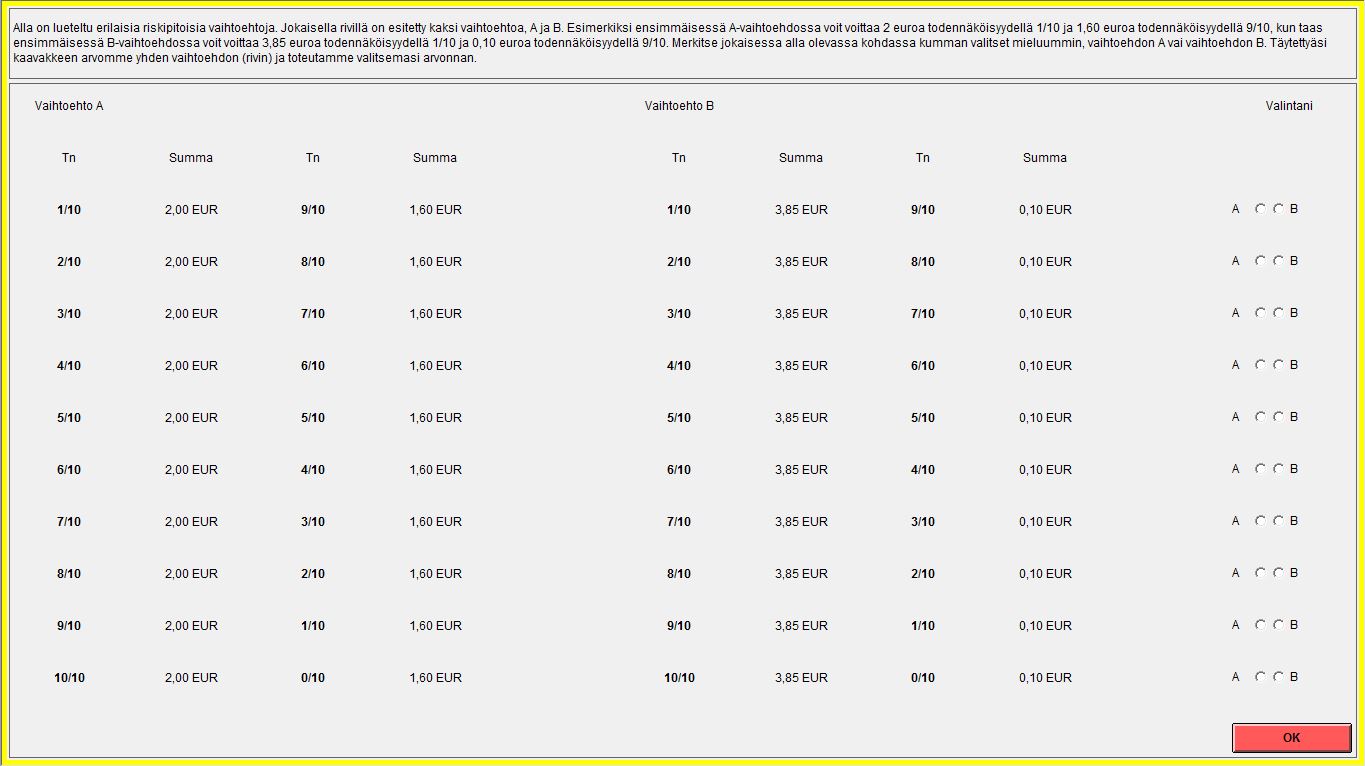


**Figure S10:** Holt-Laury risk aversion measurement screen

**
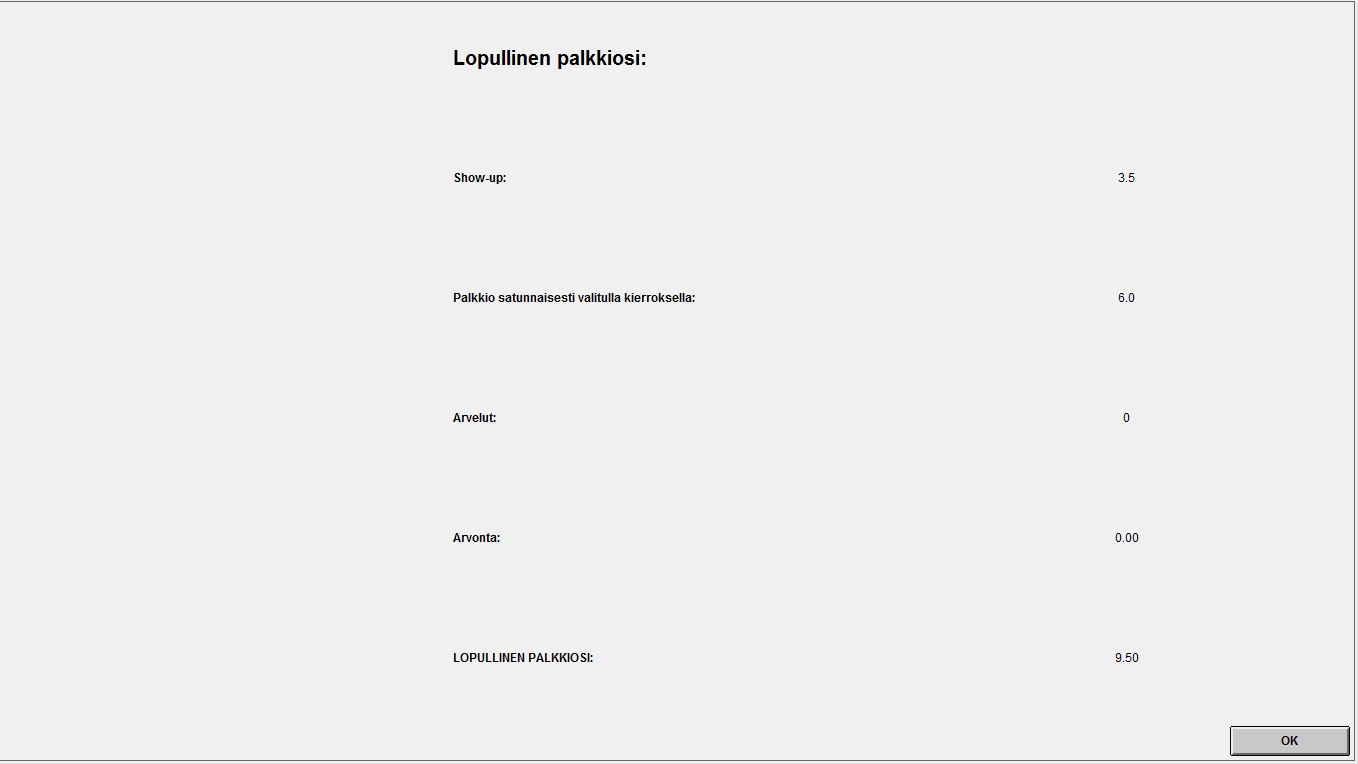
**

**Figure S11:** Final payoff announcement screen

**References**

Fischbacher, U. (2007). Z-tree, Zurich toolbox for ready-made economic experiments. *Experimental Economics*, 10, 171−178.

Greiner, B. (2015). Subject pool recruitment procedures: Organizing experiments with ORSEE. *Journal of the Economic Science Association*, 1, 114−125.
