# Supplementary material for: From ideals to deals—The effect of impartiality experience on stakeholder behavior
Source: PLoS One. 2017 Aug 7;12(8):e0182263. doi: 10.1371/journal.pone.0182263 (PMC5546632; doi:10.1371/journal.pone.0182263)
Supplement: S2 Appendix — The theoretical setup is adapted from Konow (2000) to our dynamic setting. (DOCX) [file pone.0182263.s002.docx]

**Halko, Marja-Liisa & Miettinen, Topi: From ideals to deals - the effect of impartiality experience on stakeholder behavior**

**S2 File. Theory.** The theoretical setup is adapted from Konow (2000) to our dynamic setting.

Konow (2000) presents a theory of fairness and cognitive dissonance. Applied to our context, stakeholder behavior is motivated by self-interest and fairness and the avoidance of cognitive dissonance. Applying Konow's theory, the stakeholder's maximization problem when choosing how much to allocate to oneself in the dictatorial allocation decision can be written as^[[1]](#footnote-1)^

${max}_{\phi,y}\left\{ \left( 1-\alpha_{i} \right)\left( 1-\beta_{i} \right)y-\frac{\alpha_{i}}{2}\left( y-\phi\right)^{2}-\frac{\beta_{i}}{2}\left( \phi-\eta\right)^{2} \right\}$ (1)

where $y$ is the amount allocated to oneself, $\phi$ is the belief regarding the fair entitlement, and $\eta$ is the average impartial observer view of a fair entitlement, $\alpha_{i}$ and $\beta_{i}$ are individual preference parameters capturing sensitivities to fairness and conformity, respectively. The utility function exhibits conflicting goals between bringing about outcomes that are favorable to oneself (self-interest) and outcomes that are fair. This conflict implicates a tension which is the scope of the “cognitive dissonance” literature in social psychology [Festinger, 1957] and modelled and studied by economists such as Akerlof and Dickens [1982], Oxoby [2003]. The agent strives to reduce this tension by either reducing self-interested behavior or by engaging in self-serving management of one's own beliefs about what is fair (which may well be taking place somewhat unconsciously). This latter option is modelled as a rational choice of one's own view of what is fair, $\phi$. Yet, there will be dissonance also if that view differs drastically from the impartial view denoted by $\eta$.

A third party arbitrator faces a different problem where she has no own stake in the problem and thus the maximization problem is written as

${max}_{\phi,y}\left\{ -\frac{\alpha_{i}}{2}\left( y-\phi\right)^{2}-\frac{\beta_{i}}{2}\left( \phi-\eta\right)^{2} \right\}$,

where $y$ is now the share allocated by the third party to the stakeholder in the role corresponding to the decision maker in (1). The impartial arbitrators who do not have any personal stake, choose optimally $y^{*}=\phi^{*}=\eta$.

Stakeholders’ decisions depend on their personal parameters. In general, they trade off the marginal material benefit against moral costs of unfairness and self-deception. The optimal interior solution satisfies $y^{**}=\frac{\left( 1-\alpha_{i} \right)\left( 1-\beta_{i} \right)}{\alpha_{i}}+\phi$ and $\phi^{**}=\frac{\alpha_{i}y+\beta_{i}\eta}{\alpha_{i}+\beta_{i}}$ and thus

$$y^{**}=\frac{\left( 1-\beta_{i} \right)}{\alpha_{i}}\frac{\left( 1-\alpha_{i} \right)\left( \alpha_{i}+\beta_{i} \right)}{\beta_{i}}+\eta$$

$$\phi^{**}=\frac{\left( 1-\alpha_{i} \right)\left( 1-\beta_{i} \right)}{\beta_{i}}+\eta$$

which are both larger than $\eta$. Thus, the stakeholder optimally deceives herself and self-servingly inflates her perception about her fair entitlement which allows her to grab an even larger share at the same time keeping check of the moral cost of unfairness.

When it comes to Hypothesis 1, the outcome variable we study is $\left| \phi_{i,t}-\bar{\eta}_{1} \right|$ where $\phi_{i,t}$ is the individual *i* arbitrator allocation to the poor in period *t* and $\bar{\eta}_{1}$ is the allocation of an average arbitrator in the first period (6.94 in our sample, N = 98). The absolute value is taken since the rich and the poor bias their allocation to the poor in opposite directions. In Hypothesis 2, the outcome variable we study is $\left| y_{i,t}-\bar{\eta}_{1} \right|$ where $y_{i,t}$ is the stakeholder’s allocation to the poor. In Hypothesis 3, the outcome variable we study is $\left| d_{i,t}-\bar{\eta}_{1} \right|$ where $d_{i,t}$ is the stakeholder Nash demand allocation to the poor (in the case of rich stakeholders $d_{i,t}=12-x_{i,t}$ where $x_{i,t}$ is how much the rich stakeholder demands for herself; in the case of poor stakeholders $d_{i,t}=x_{i,t}$)

**References**

Akerlof, G.A. & Dickens, W.D. (1982). The economic consequences of cognitive dissonance. *American Economic Review*, 72:307−19.

Festinger, L. (1957). A theory of cognitive dissonance. Row and Peterson, Evanston, IL.

Konow, J. (2000). Fair shares: accountability and cognitive dissonance in allocation decisions. *American Economic Review*, 90, 1072−1091.

Oxoby. R.J. (2003). Attitudes and allocations: Status, cognitive dissonance, and the manipulation of preferences*. Journal of Economic Behavior and Organization*, 52:365−85.

1. Konow [2000] presents a version with more general functional forms but for our purposes the quadratic formulation serves our illustrative purposes. The results generalize in a straightforward manner. [↑](#footnote-ref-1)
