# Supplementary material for: From ideals to deals—The effect of impartiality experience on stakeholder behavior
Source: PLoS One. 2017 Aug 7;12(8):e0182263. doi: 10.1371/journal.pone.0182263 (PMC5546632; doi:10.1371/journal.pone.0182263)
Supplement: S3 Appendix — (DOCX) [file pone.0182263.s003.docx]

**Halko, Marja-Liisa & Miettinen, Topi: From ideals to deals - the effect of impartiality experience on stakeholder behavior**

## **S4 File: Supportive evidence and regressions**

In Table S4.1 below, we regress the Nash demand decisions of the stakeholders on (i) the stakeholder role, (ii) their guesses or beliefs about other party's behavior, (iii) and on the individual risk aversion measure. Regression models 1-3 regress the negotiation behavior of the respective period. The bench-line is the stakeholder behavior of the participant in role A. Thus the first model studies stakeholders without arbitration experience, the second model examines those with arbitration experience, and the last column has the data from the same participants as in the first model, but now when they have played the interaction once in the role of the stakeholder and once in the role of the arbitrator.

There is no difference in the Nash-demands of the two negotiating parties in the first period, not even when controlling for risk-aversion and beliefs. In the second period, the ex-arbitrators ending up in the role of the poor stakeholder make a higher Nash-demand and the demand is correlated with what the Bs believe other participants in the same role are doing (the interaction between Role B and Guess b). Since we do not have independent exogenous (experimental) variation in beliefs, one cannot say whether this is evidence that conformism matters. Alternatively, a positive association between own behavior and beliefs about others' behavior in a similar situation could be brought about by consensus bias in beliefs (Blanco et al., 2014; Ross et al. 1977), for instance, i.e. a potentially false belief that others' behavior is more similar to one's own behavior than it actually is. Notice also that there is a similar positive association between the beliefs and the behavior of As (variable Guess a). The beliefs regarding the behavior of the opponents is negatively associated with the Nash demands (variable Guess b and the interaction between Role B and Guess a). This is in line with strategic best-response behavior. Yet similar words of caution apply here: the direction of the causal effect cannot be identified due to the lack of exogenous variation. Also the role of risk-aversion is line with the theoretical prediction: Nash demands of the risk-averse are smaller in the first period. Yet the effect is insignificant at the latter two periods.

| **Table S4.1. Nash demand decisions of the stakeholders.** | | | |
| --- | --- | --- | --- |
| VARIABLES | (1)  Nash-demand | (2)  Nash-demand | (3)  Nash-demand |
| Role B (poor) | -1.072  (1.285) | 3.432*  (1.851) | 0.0684  (1.388) |
| Guess a | 0.357***  (0.091) | 0.618***  (0.180) | 0.454***  (0.098) |
| Role B × Guess a | -0.471***  (0.150) | -0.865***  (0.213) | -0.628***  (0.162) |
| Guess b | -0.189**  (0.093) | -0.0710  (0.145) | -0.434***  (0.101) |
| Role B × Guess b | 0.653***  (0.135) | 0.403**  (0.176) | 0.719***  (0.146) |
| H&L-score | -0.0984*  (0.055) | -0.0670  (0.0734) | -0.0888  (0.059) |
| Constant | 5.442***  (1.030) | 2.357  (1.779) | 5.848***  (1.112) |
| Observations | 78 | 74 | 78 |
| R-squared | 0.498 | 0.513 | 0.625 |
| Linear regression models on stakeholders’ Nash-demands in periods one, two and three. Standard errors in parentheses, *** p<0.01, ** p<0.05, * p<0.1 | | | |

Let us then turn to a regression analysis of the arbitration decisions by the third parties. In Table S4.2 the bench-line in the regressions is the behavior of arbitrator C and the dummy variable “role D” captures the difference between the arbitrators in roles D and C. The table illustrates that there is little difference in the arbitration decisions between the Cs and the Ds in the first period. This is merely a check that there are no labelling effects in the arbitration decisions and that the randomization of the participants to different roles has been successful.

| **Table S4.2. Dictatorial decisions of the arbitrators in period one.** | | |
| --- | --- | --- |
| VARIABLES | (1)  Dictatorial allocation | |
| Role D | -0.953  (2.631) | |
| Guess a | -0.302  (0.205) | |
| Role D × Guess a | 0.385  (0.256) | |
| Guess b | 0.331  (0.225) | |
| Role D × Guess b | -0.138  (0.273) | |
| Constant | 6.190***  (2.244) | |
| Observations | 85 | |
| R-squared | 0.114 | |
| Linear regression models on arbitrators’ dictatorial allocations in period one. Standard errors in parentheses, *** p<0.01, ** p<0.05, * p<0.1. | |  |

Linear regression models 1 and 2 in Table S4.3 study how the arbitration decisions of the arbitrators in the C and D role, respectively, are impacted by negotiation experience. Consider first Model 1. In Period 2, the rich stakeholders become arbitrators. Now that we control for beliefs, the upward shift from period 1 to 2 in the amount assigned to the poor is not significant. In fact, none of the explanatory variables has a significant impact on the share of the poor stakeholder.

| **Table S4.3. Dictatorial decisions of the arbitrators in period two and three.** | | | |
| --- | --- | --- | --- |
| VARIABLES | (1)  Dictatorial allocation | (2)  Dictatorial allocation | |
| Ex-rich / ex-poor | 1.670  (2.884) | 6.680***  (1.887) | |
| Guess a | -0.302  (0.205) | 0.083  (0.134) | |
| Ex-rich / ex-poor × Guess a | -0.0118  (0.267) | -0.940***  (0.235) | |
| Guess b | 0.331  (0.226) | 0.193  (0.136) | |
| Ex-rich / ex-poor × Guess b | -0.189  (0.289) | -0.236  (0.206) | |
| Constant | 6.190***  (2.251) | 5.238***  (1.208) | |
| Observations | 90 | 86 | |
| R-squared | 0.114 | 0.227 | |
| Linear regression models on arbitrators’ dictatorial allocations in the first two periods. Standard errors in parentheses, *** p<0.01, ** p<0.05, * p<0.1. | | |  |

Among the ex-poor (Model 2 in Table S4.3) in the second period, the arbitrators' beliefs regarding how much a rich stakeholder demands in the negotiations (variable Guess a) are negatively associated with the amount assigned to poor. There is no such effect in the first period. Again the causal effect in the second period is unclear - this is merely an association. This could indicate, for instance, that an arbitrator's fairness ideal regarding the fair share to A is positively correlated with her guess regarding As' demands (which should be the case if the ex-poor stakeholders think it at all likely that fairness at all influences As' demands) and likewise negatively correlated with how much the arbitrator gives to a poor stakeholder (which should be the case if fairness matters at all to the arbitrator in question). The median arbitrator in the D role (ex-poor) estimates the demand of As at 6 (half the pie) in the second period. Therefore, a D-arbitrator with a median guess about As' demands will assign 6.3 euros to the poor while a D-arbitrator with a guess at the 2nd decile at 5, will assign 7.24 euros to the poor stakeholder.

The effect of the second period (negotiation experience) is highly significant among the arbitrators who were poor stakeholders in the preceding round. The linear regression coefficient of 6.68 applies to those who guess that As and Bs ask for nothing. These constitute very extreme beliefs; so one may wish to calculate the implied predicted marginal effect of negotiation experience when beliefs are average beliefs. Holding beliefs constant at the average beliefs, the marginal effect of negotiation experience in the poor stakeholder role alone is negative at -0.44 euros and insignificantly different from zero but, indeed, the ex-poor arbitrators who guess the rich stakeholders to assign a low share to the poor also assign a lower share to the poor (significant interaction 2.period x Guess a).

**References**

Blanco M, Engelmann D, Koch AK & Normann HT. Preferences and beliefs in a sequential social dilemma: a within-subjects analysis. Games and Economic Behavior. 2014; 87, 122−135.

Ross L, Greene D & House P. The “false consensus effect”: An egocentric bias in social perception and attribution processes. Journal of experimental social psychology. 1977; 13(3), 279−301.
