# Supplementary material for: From ideals to deals—The effect of impartiality experience on stakeholder behavior
Source: PLoS One. 2017 Aug 7;12(8):e0182263. doi: 10.1371/journal.pone.0182263 (PMC5546632; doi:10.1371/journal.pone.0182263)
Supplement: S1 Table — Linear regression models on stakeholder’ Nash-demands and dictatorial allocations in the first two periods. (DOCX) [file pone.0182263.s004.docx]

**Halko, Marja-Liisa & Miettinen, Topi: From ideals to deals - the effect of impartiality experience on stakeholder behavior**

**S3 Table. Difference-in-differences tests.**

| Linear regression models on stakeholder’ Nash-demands and dictatorial allocations in the first two periods | | | |
| --- | --- | --- | --- |
| VARIABLES | (1)  Nash-demand | (2)  Dictatorial allocation | |
| Poor | 0.664**  (0.291) | 2.304***  (0.541) | |
| Arbitration experience | -0.683**  (0.293) | 0.105  (0.544) | |
| Poor × Arbitration experience | 0.925**  (0.419) | 0.725  (0.778) | |
| Constant | 5.404***  (0.203) | 5.128***  (0.376) | |
| Observations | 176 | 176 | |
| R-squared | 0.165 | 0.221 | |
| Standard errors in parentheses, *** p<0.01, ** p<0.05, * p<0.1 | | |  |
